# Degradation kinetics of vitamins in premixes for pig: effects of choline, high concentrations of copper and zinc, and storage time

**DOI:** 10.5713/ajas.20.0026

**Published:** 2020-06-24

**Authors:** Pan Yang, Hua Kai Wang, Min Zhu, Long Xian Li, Yong Xi Ma

**Affiliations:** 1 State Key Laboratory of Animal Nutrition, College of Animal Science and Technology, China Agricultural University, Beijing 100193, China; 2 Feed Research Center, Liyuan Group, Guangxi 541004, China; 3 Ministry of Agriculture and Rural Affairs Feed Industry Centre, Beijing 100193, China

**Keywords:** Choline, Copper, Premix, Vitamin Stability, Storage Time, Zinc

## Abstract

**Objective:**

The present work was undertaken to evaluate the effects of storage time, choline chloride, and high concentrations of Cu and Zn on the kinetic behavior of vitamin degradation during storage in two vitamin premixes and four vitamin-trace mineral (VTM) premixes.

**Methods:**

Two vitamin premixes (with or without 160,000 mg/kg of choline) were stored at 25°C and 60% humidity. Besides, four VTM premixes were used to evaluate the effects of choline (0 vs 40,000 mg/kg) and trace minerals (low CuSO_4_+ZnO vs high CuSO_4_+ZnO) on vitamin stability in VTM premixes stored in room, and the VTM premixes were stored in room temperature at 22°C. Subsamples from each vitamin and VTM premix were collected at 0, 1, 2, 3, 6, and 12 months. The retention of vitamin A (VA), vitamin D_3_ (VD_3_), vitamin E (VE), vitamin K_3_ (VK_3_), vitamin B_1_ (VB_1_), vitamin B_2_ (VB_2_), vitamin B_3_ (VB_3_), vitamin B_5_ (VB_5_), and vitamin B_6_ (VB_6_) in vitamin premixes and VTM premixes during storage was determined. The stability of vitamins in vitamin premixes and VTM premixes was determined and reported as the residual vitamin activity (% of initial) at each sampling point.

**Results:**

The effect of choline on VK_3_ retention was significant in vitamin premixes (p<0.05). The negative effect of storage time was significant for the retentions of VD_3_, VK_3_, VB_1_, VB_2_, VB_5_, and VB_6_ in vitamin premix (p<0.05). For VTM premixes, negative effect of storage time was significant (p<0.05) for the losses of vitamin in VTM premixes. Choline and high concentrations of Cu and Zn significantly increased VA, VK_3_, VB_1_, and VB_2_ loss during storage (p<0.05). The supplementation of high concentrations of Cu and Zn significantly decreased the concentrations of VD_3_ and VB_6_ (p<0.05) in VTM premixes at extended storage time.

**Conclusion:**

The maximum vitamin stability was detected in vitamin and VTM premixes containing no choline or excess Cu and Zn. The results indicated that extended storage time increased degradation of vitamin in vitamin or VTM premixes. These results may provide useful information for vitamin and VTM premixes to improve the knowledge of vitamin in terms of its stability.

## INTRODUCTION

Vitamin and vitamin-trace mineral (VTM) premixes are designed for supplemental nutritional support to animals who are unable to ingest adequate amounts of natural feedstuffs [1,2]. Choline serves some essential biological functions in young animals [3,4], such as improving fat transport and metabolism in the liver, source of methyl donors for methionine regeneration from homocysteine, building and maintaining cell wall structure, and supporting nervous system function.

In the post-weaning period, the stress of being removed from the sow and mixing into a new environment results in perturbations of gut microbiota and lowered defenses against pathogen entry for piglets, which can lead to increased risk of disease [5,6]. Post-weaning diarrhea is one of the most common causes of mortality for weanling pigs, and hence greatly impaired growth performance of pigs [5,6]. In practical application, the inclusion of high concentrations of zinc oxide (ZnO) and copper sulfate (CuSO_4_) in weaned pig diets can improve the growth performance and decrease diarrhea incidence [5–7]. All of these treatments may affect the formulation of the ingredients in premixes, leading to bioavailability loss of necessary nutrients. In addition, premixes are often not consumed immediately after manufacturing and can be degraded due to several factors, such as the length of storage and premix compositions. (e.g. choline chloride and high concentrations of metal ions) [3,8–11]. Numerous reports have discussed the stability of vitamins under various conditions such as storage and processing [8–11]. Vitamins may be unstable to heat and light as well as exposure to acid, alkali, air, and moisture. The degradation of vitamin activity in premixes and complete feed during storage may result in hidden depressions in growth, feed efficiency, and disease resistance due to the subclinical vitamin deficiencies [3,8,11]. For this reason, the vitamin content should be evaluated after manufacturing and storage in order to ensure the amount provided.

However, studies on the stability of vitamins in vitamin premix and VTM premix are limited. In addition, these factors (choline chloride and high concentrations of CuSO_4_ and ZnO) in premixes have received limited research attention, and it is unclear which vitamins in vitamin or VTM premixes are vulnerable when choline and high concentrations of Cu and Zn are present. Therefore, the objectives of this study were to i) determine the rate of vitamin loss in vitamin or VTM premixes and the effects of choline and high concentrations of Cu and Zn on the stability of vitamins during storage and ii) to develop vitamin retention prediction models based on storage time.

## MATERIALS AND METHODS

### Experimental design

This study was conducted at the State Key Laboratory of Animal Nutrition at China Agricultural University (Beijing, China) and Ministry of Agriculture and Rural Affairs Feed Efficacy and Safety Evaluation Center (Beijing, China). Approval from the Animal Care and Use Committee was not obtained for this experiment because no animals were used.

### Chemical reagents

Deionized water (18 Mω·cm) from a Millipore Milli-Q (Bedford, MA, USA) water purification system was used to prepare all aqueous solutions. Standard for retinyl esters, cholecalciferol, α-tocopherol acetate, menadione, thiamine, riboflavin, pyridoxine, niacin and pantothenic acid were purchased from Sigma–Aldrich (Fluka, Sigma–Aldrich, Steinheim, Germany). The methanol and acetonitrile of high-performance liquid chromatography (HPLC) grade were used for HPLC analysis and were obtained from Fisher Scientific (Pittsburg, PA, USA). All the other chemicals used were analytical grade and purchased from Sinopharm Chemical Reagent LTD (Beijing, China).

### Premix formulation and treatments

Two vitamin premixes (containing no trace minerals) were formulated by commercial vitamins. The vitamin manufacturer is not disclosed in order to protect proprietary information. The manufacturing dates of all vitamins were obtained from the original suppliers to ensure that the products were within 6 months of manufacture and were not expired. The vitamin premixes were designed to be added at a rate of 2.5 g/kg of the diet. The specific vitamin composition and inclusion level were set to mimic common swine industry vitamin premixes. One of vitamin premixes contained choline, another vitamin premix without choline (Table 1). The choline chloride concentration in vitamin premix was measured *via* chromatography [12].

Four VTM premixes were formulated to contain the same level of vitamins for weanling piglets. The VTM premixes were designed to be added at a rate of 10 g/kg of the diet, which is common in practice. Compositions of VTM premixes were shown in Table 1. Vitamin levels met or exceeded the requirement of NRC [13], which were chosen to represent the “typical” industry levels based on informal surveys of vitamin levels in commercially available premixes. The amounts of each vitamin used in each premix are shown in Table 2. Choline was added to VTM premixes 2 and 4. The choline chloride concentration in VTM premix was determined using chromatography [12]. The VTM premixes 1 to 4 were formulated to meet or exceeded NRC requirements for copper (Cu), iodine (I), iron (Fe), manganese (Mg), selenium (Se), and zinc (Zn) for piglets [13]. The VTM premixes 3 and 4 contained 20,000 mg/kg of Cu added as CuSO_4_ and 225,000 mg/kg per diet of Zn added as ZnO, which could provide 200 mg/kg of Cu and 2,250 mg/kg Zn in diet. The reason for choosing these levels of Cu and Zn is that weanling pig premixes commonly have higher concentrations of Cu and Zn as an antimicrobial and improve growth performance [5–7].

### Premix preparation and storage

Vitamin and VTM mineral premixes were manufactured at a commercial vitamin premix plant. Each of the two vitamin premixes was prepared in six separate batches using an identical procedure (7.2 kg per batch). Each batch represented one replicate and was divided into six-1.2 kg, thick polyethylene bags with plastic ties. Each of the four VTM premixes was prepared in six separate and identical procedure (18 kg per batch). Each batch represented one replicate and was divided into six-3.0 kg, thick polyethylene bags with plastic ties. Vitamin premixes were stored in a controlled environment chamber setting at 25°C and 60% relative humidity. The VTM premixes were stored in a storage room (22°C).

### Vitamin sampling, extraction and assays

Subsample of vitamin and VTM premixes were obtained from each of six replicates at 0, 1, 2, 3, 6, and 12 months. Samples were immediately sent to the Ministry of Agriculture and Rural Affairs Feed Efficacy and Safety Evaluation Center (Beijing, China) used for vitamin analysis. The vitamin A (VA) and E (VE) were determined by the method 2012.10 [14]. In brief, the sample (2 g) was mixed with papain solution until dispersed, placed in a 37°C±2°C water bath, and extracted by methanol. This extract was analyzed by HPLC (Agilent 1200 Series; Agilent Technologies Inc., Santa Clara, CA, USA). For the extraction of vitamin D_3_ (VD_3_) from samples, the method of 992.26 [14] was used. In brief, 5 g sample was transferred to a centrifuge tube and anhydrous ethanol, ascorbic acid, and potassium hydroxide were added. Tubes were placed in a 75°C water bath. Subsamples were analyzed by HPLC followed by UV detection at 254 nm. For the determination of vitamin K_3_ (VK_3_), samples were extracted with trichloromethane. Extract filtered and injected into the HPLC system and UV detection was made at wavelength 251 nm [15]. For the extraction of water-soluble vitamins (vitamin B_1_ [VB_1_], vitamin B_2_ [VB_2_], vitamin B_3_ [VB_3_], vitamin B_5_ [VB_5_], and vitamin B_6_ [VB_6_]) from diets, the procedure of Chen et al [16] was used and modified. Five g of sample was weighed, extracted with phosphate buffer, heated in a water bath, and sonicated. The supernatants of the extracted samples were stored at −20°C until they were tested. These extracted samples were analyzed using a 250× 4.5 mm, 5 μm, Eclipse Plus C18 column (Agilent Technologies Inc., USA) on an Agilent liquid chromatograph. The stability of vitamins in vitamin premixes and VTM premixes during storage was determined and reported as the residual vitamin activity (% of initial) at each sampling point. This time was also convenient for us to compare with previous studies and for developing predicted equations to estimate the vitamin loss. The specific vitamin selected were based on the capacity to complete our respective lab analysis.

### Data treatment and statistical analysis

Normality of the data was verified using the UNIVARIATE procedure of SAS (SAS Inst. Inc., Cary, NC, USA). The BOXPLOT procedure of SAS was used to check for outliers. Data were analyzed using the MIXED procedure of SAS (SAS Inst. Inc., USA) to determine the interactive and main effects of choline chloride, high concentrations of Zn and Cn, and storage time on the activity of vitamins in vitamin and VTM premixes. Results were considered significant at p≤0.05 and a tendency at p≤0.10. Diagrams were generated using the Excel 2016 (Microsoft Corporation, Redmond, WA, USA). Vitamin retention was modelled using linear regression and non-linear regression. Linear regression to determine vitamin retentions was completed using the PROC REG procedure of SAS. Non-linear regression was performed using PROC NLIN procedures of SAS with exponential model (Eq. (1)). The exponential model is flexible owing to the inclusion of a shape constant in addition to the rate constant and has been employed to describe vitamin degradation kinetics [17].

(1) $$C_{t}=C_{0}\times e^{-K_{aa}\;t}$$

Where *C_t_* is the vitamin concentration at a time *t*, *C*_0_ is the initial vitamin concentration, *K_aa_* is the rate constant. Modelling and analysis of variance were performed using SAS 9.4 (SAS Inst. Inc., USA). The R^2^ and the root mean square error of prediction (RMSEP) were used to define the best-fit equations.

## RESULTS

### Vitamin recovery method validation

Methods for vitamin analysis in samples were validated with repeatability between-day precision, long-term precision, limits of quantitation, and linearity (data not shown) by the staff of the Ministry of Agriculture and Rural Affairs Feed Efficacy and Safety Evaluation Center (Beijing, China). Calculated values (Table 1) were determined from the minimum declared vitamin concentrations as provided by each product’s manufacturer. The initial (d 0) analyzed vitamin concentrations of the vitamin premix, or VTM premixes are reported in Table 2. The analyzed vitamin values in vitamin premix or VTM premixes were similar to calculated vitamin values in vitamin premix or VTM premixes.

### Effects of storage time and choline on stability of vitamins in vitamin premixes

**Figure 1:**
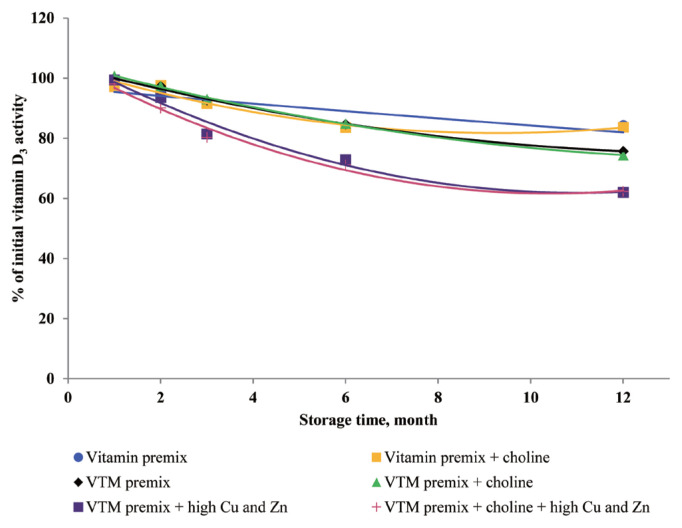
Residual vitamin D_3_ activity (% of initial) for vitamin premixes and vitamin-trace mineral (VTM) premixes as affected by choline chloride, high concentrations of copper (Cu) and zinc (Zn) and time (1 to 12 months). Each data point is the mean of 6 observations. The p-values in vitamin premix (time×choline, NS; time, p<0.001; choline, NS). The p-values in VTM premix (time×choline×Cu/Zn, NS; time× choline, NS; time×Cu/Zn, p<0.001; choline×Cu/Zn, NS; time, p<0.001; choline, NS; Cu/Zn, p<0.001).

**Figure 2:**
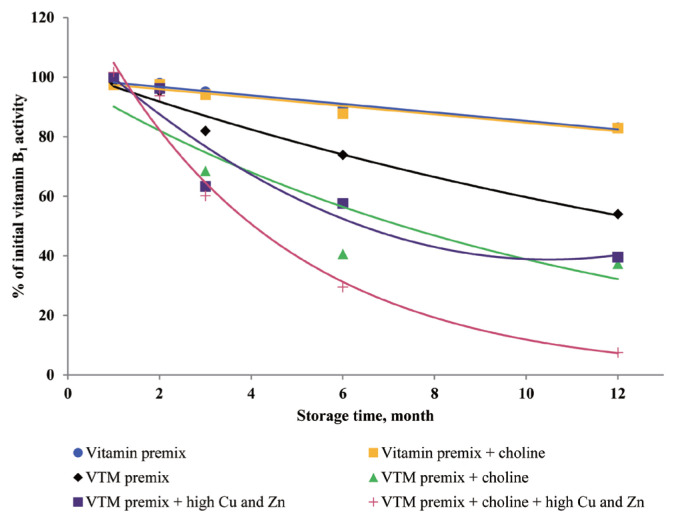
Residual vitamin B_1_ activity (% of initial) for vitamin premixes and vitamin-trace mineral (VTM) premixes as affected by choline chloride, high concentrations of copper (Cu) and zinc (Zn) and time (1 to 12 months). Each data point is the mean of 6 observations. The p-values in vitamin premix (time×choline: NS; time, p<0.001; choline, NS). The p-values in VTM premix (time×choline×Cu/Zn, p = 0.013; time×choline, p<0.001; time×Cu/Zn, p<0.001; choline×Cu/Zn, NS; time, p<0.001; choline, p<0.001; Cu/Zn, p<0.001).

**Figure 3:**
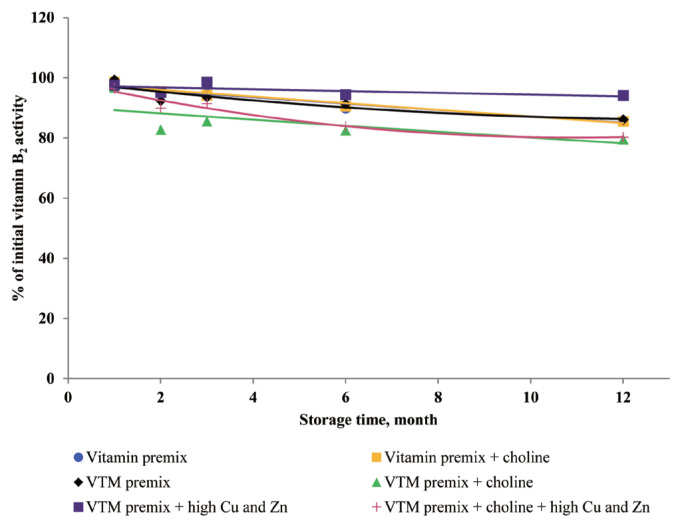
Residual vitamin B_2_ activity (% of initial) for vitamin premixes and vitamin-trace mineral (VTM) premixes as affected by choline chloride, high concentrations of copper (Cu) and zinc (Zn) and time (1 to 12 months). Each data point is the mean of 6 observations. The p-values in vitamin premix (time×choline, NS; time, p<0.001; choline, NS). The p-values in VTM premix (time×choline×Cu/Zn, p = 0.009; time×choline, p<0.001; time×Cu/Zn, p = 0.001; choline×Cu/Zn, NS; time, p<0.001; choline, p<0.001; Cu/Zn, p<0.001).

**Figure 4:**
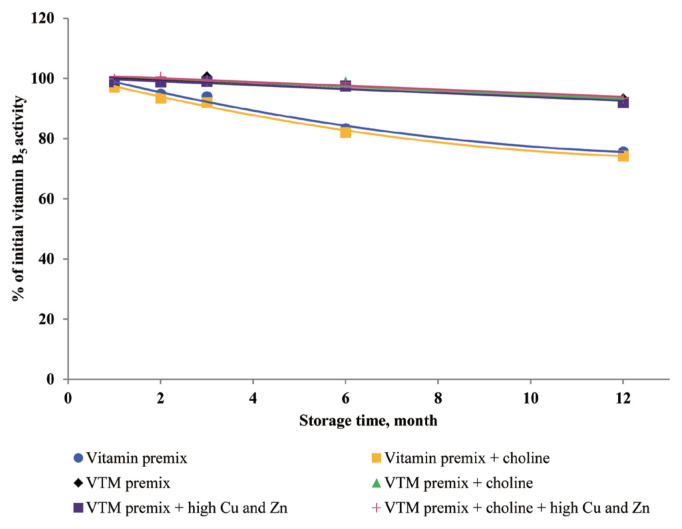
Residual vitamin B_5_ activity (% of initial) for vitamin premixes and vitamin-trace mineral (VTM) premixes as affected by choline chloride, high concentrations of copper (Cu) and zinc (Zn) and time (1 to 12 months). Each data point is the mean of 6 observations. The p-values in vitamin premix (time×choline, NS; time, p<0.001; choline, p = 0.053). The p-values in VTM premix (time×choline×Cu/Zn, NS; time×choline, NS; time×Cu/Zn, NS; choline×Cu/Zn, NS; time, p<0.001; choline, NS; Cu/Zn, NS).

**Figure 5:**
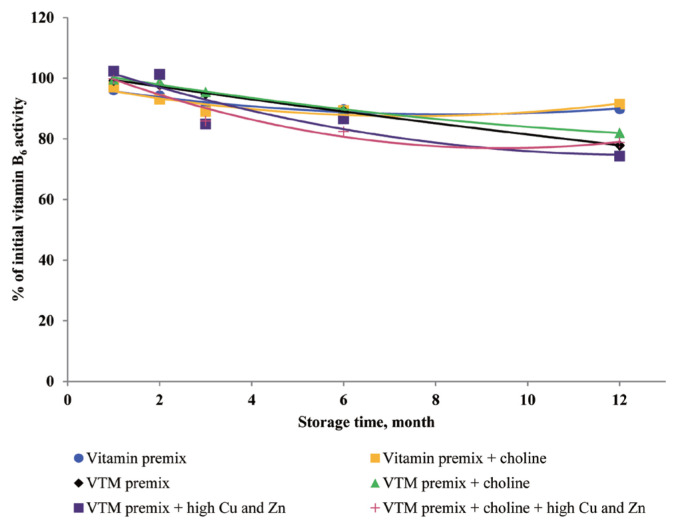
Residual vitamin B_6_ activity (% of initial) for vitamin premixes and vitamin-trace mineral (VTM) premixes as affected by choline chloride, high concentrations of copper (Cu) and zinc (Zn) and time (1 to 12 months). Each data point is the mean of 6 observations. The p-values in vitamin premix (time×choline, NS; time, p<0.001; choline, NS). The p-values in VTM premix (time×choline×Cu/Zn, NS; time× choline, p = 0.052; time×Cu/Zn, p<0.001; choline×Cu/Zn, NS; time, p<0.001; choline, NS; Cu/Zn, p<0.001).

**Figure 6:**
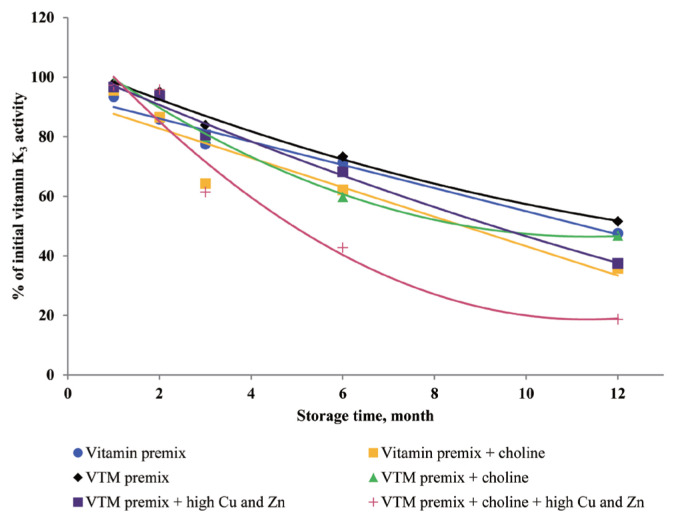
Residual vitamin K_3_ activity (% of initial) for vitamin premixes and vitamin-trace mineral (VTM) premixes as affected by choline chloride, high concentrations of copper (Cu) and zinc (Zn) and time (1 to 12 months). Each data point is the mean of 6 observations. The p-values in vitamin premix (time×choline, p<0.001; time, p<0.001; choline, p<0.001). The p-values in VTM premix (time×choline×Cu/Zn, p = 0.002; time×choline, p<0.001; time×Cu/Zn, p<0.001; choline×Cu/Zn, p<0.001; time, p<0.001; choline, p<0.001; Cu/Zn, p<0.001).

There was no significant interactive effect of storage time and choline on VA, VD_3_, VE, VB_1_, VB_2_, VB_3_, VB_5_, and VB_6_ retention (Table 3). The main effect of storage time was significant (p<0.01) for the retentions of VD_3_, VB_1_, VB_2_, VB_5_, and VB_6_ in vitamin premix (Figures 1 to 5). The retention of VK_3_ was significantly influenced by storage time (p<0.01), choline (p<0.01) and the interaction between storage time and choline (p<0.01; Table 3; Figure 6).

At 3 months, VA, VD_3_, VE, VK_3_, VB_1_, VB_2_, VB_3_, VB_5_, and VB_6_ retained at least 96%, 92%, 97%, 78%, 95%, 94%, 98%, 94%, and 90% of their initial activity in vitamin premix without choline, respectively. At 12 months, most vitamins retained 76% to 98% of the initial vitamin activity, but VK_3_ was retained 47.64% of its initial activity in vitamin premix without choline. With increased storage time, VA exhibited a tendency (p = 0.096) for decreased retention in vitamin premixes, but VA exhibited a tendency (p = 0.076) and VB_5_ was marginally significant (p = 0.053) for decreased retention during extended storage time in vitamin premixes containing choline chloride.

### Effects of storage time, choline, and Cu and Zn elements on stability of vitamins in VTM premixes

**Figure 7:**
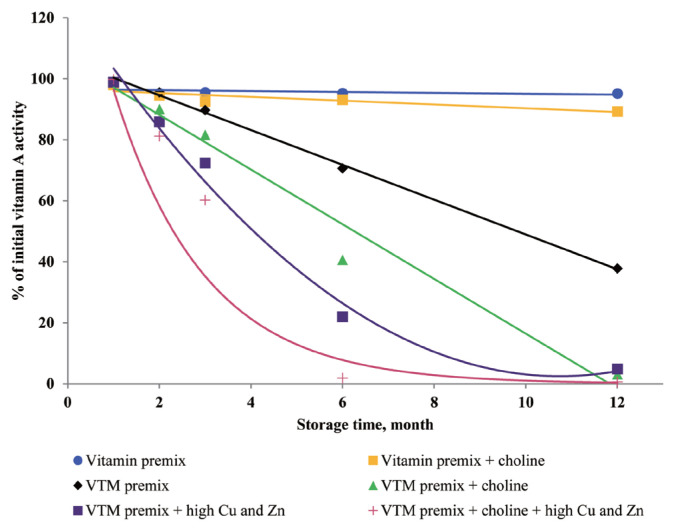
Residual vitamin A activity (% of initial) for vitamin premixes and vitamin-trace mineral (VTM) premixes as affected by choline chloride, high concentrations of copper (Cu) and zinc (Zn) and time (1 to 12 months). Each data point is the mean of 6 observations. The p-values in vitamin premix (time×choline, NS; time, NS; choline, NS). The p-values in VTM premix (time×choline×Cu/Zn, NS; time×choline, p = 0.011; time×Cu/Zn, p<0.001; choline×Cu/Zn, NS; time, p<0.001; choline, p<0.001; Cu/Zn, p<0.001).

There were interactive effects among storage time, choline chloride and high concentrations of Cu and Zn on VK_3_, VB_1_, and VB_2_ retentions (p<0.05) (Table 4). Moreover, there were interactive effects between storage time and choline chloride on VA, VK_3_, VB_1_, and VB_2_ retentions (p<0.05). There were interactive effects between storage time and high concentrations of Cu and Zn on VA, VD_3_, VK_3_, VB_1_, VB_2_, and VB_6_ retentions (p<0.01). As well, there were interactive effects between choline chloride and high concentrations of Cu and Zn on VK_3_ retention (p<0.01). The main effect of storage time on all tested vitamins was significant (p<0.01), the main effect of choline was significant for VA, VK_3_, VB_1_, and VB_2_ retentions (p<0.01) (Figures 2, 3, 6, and 7), and the main effect of high concentrations of Cu and Zn on VA, VD_3_, VK_3_, VB_1_, VB_2_, VB_6_ retentions was significant (p<0.01) (Figures 1, 2, 3, 5, 6, and 7).

**Figure 8:**
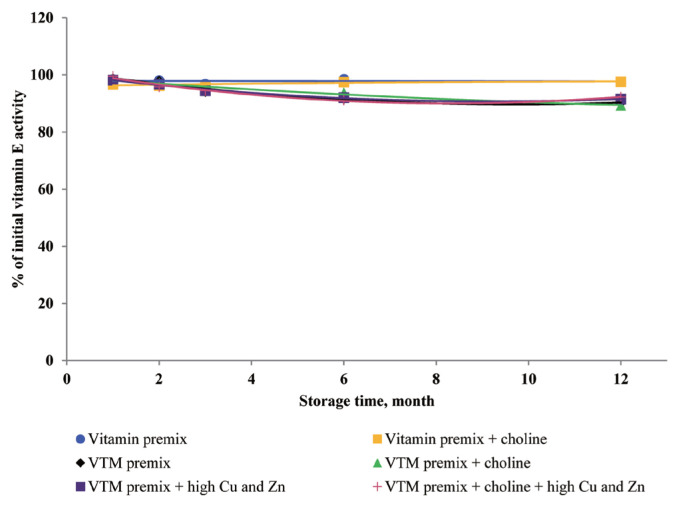
Residual vitamin E activity (% of initial) for vitamin premixes and vitamin-trace mineral (VTM) premixes as affected by choline chloride, high concentrations of copper (Cu) and zinc (Zn) and time (1 to 12 months). Each data point is the mean of 6 observations. The p-values in vitamin premix (time×choline, NS; time, NS; choline, NS). The p-values in VTM premix (time×choline×Cu/Zn, NS; time×choline, NS; time×Cu/Zn, NS; choline×Cu/Zn, NS; time, p<0.001; choline, NS; Cu/Zn, NS).

**Figure 9:**
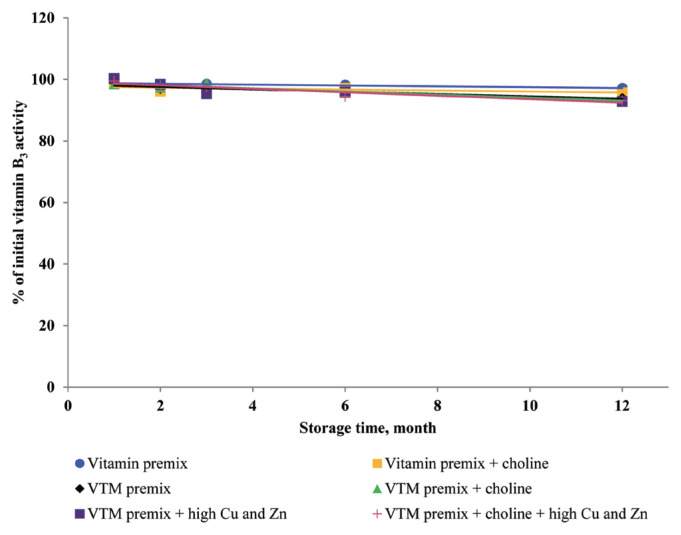
Residual vitamin B_3_ activity (% of initial) for vitamin premixes and vitamin-trace mineral (VTM) premixes as affected by choline chloride, high concentrations of copper (Cu) and zinc (Zn) and time (1 to 12 months). Each data point is the mean of 6 observations. The p-values in vitamin premix (time×choline, NS; time, NS; choline, NS). The p-values in VTM premix (time×choline×Cu/Zn, NS; time×choline, NS; time×Cu/Zn, NS; choline×Cu/Zn, NS; time, p<0.001; choline, NS; Cu/Zn, NS).

Extended storage time had negative (p<0.01) effects on vitamin stability in VTM premix. When VTM premixes were stored, VE, VB_3_, and VB_5_ activities decreased (p<0.05) (Figures 4, 8, and 9) as the duration of storage increased regardless of the choline chloride or high concentrations of Cu and Zn. Also, stability of VA, VK_3_, VB_1_, and VB_2_ was decreased in VTM premix containing choline and high concentrations of Cu and Zn. However, the activity of VB_6_ was rapidly decreased in VTM premix containing high concentrations of Cu and Zn (Figure 5).

### Kinetic properties of vitamin retention

The R^2^, RMSEP, and the prediction equations are presented (Table 5). Analysis of kinetic data suggested that the degradation follows a first-order model. Degradation of VA, VD_3_, VK_3_, VB_1_, and VB_6_ in VTM premix 1 could be suitably modeled based on high R^2^ value (i.e. R^2^>0.90) of prediction equations. For premix containing chloride choline, equations for predicting retention of VA, VD_3_, VE, VK_3_, VB_3_, and VB_6_ in VTM premix 2 were suitably modeled based on high R^2^ value (i.e. R^2^>0.90), and those had high R^2^ values compared to equations predicting VB_1_, VB_2_, and VB_5_. While the ability to predict degradation of VB_2_ with storage was low (R^2^ = 0.492). In VTM premix 3, equations for predicting retention of VA, VD_3_, VK_3_, and VB_5_ had high R^2^ values (i.e. R^2^>0.90) and thus good fit to predict change in retention with storage time. However, similar ability to predict VB_2_ degradation with storage time was low (R^2^ = 0.477). In VTM premix 4, the equations had high R^2^ (i.e. R^2^>0.90) for predicting retention of VD_3_, VK_3_, VB_1_, and VB_5_ compared to predicting other vitamins.

## DISCUSSION

### Extended storage time affected the stability of vitamins

Determination of vitamin stability before and after storing is necessary to assess the final amount of actual vitamin that will reach the end-user and to calculate what amount should be added to any feed matrix. Storing vitamins for long periods has been considered to negatively affect vitamin activity in vitamin or VTM premixes. However, the results of our study indicate that long-term storage of vitamin premixes had little influence upon VA, VE, and VB_3_ concentrations. The results from our study agreed with previous studies on vitamin stability. Coelho [8] reported that the average loss per month of VA, VE, and VB_3_ after storage was 2.9%, 2.7%, and 2.7%, respectively. Because most manufactures of VA producers stabilize VA using the method of sealing within a physical matrix, generally arabic gum or gelatin [2,18]. In addition, in the production of commercially-available VA and VE, their hydroxy group is protected by the formation of an ester, as in α-tocopherol acetate. The obtained α-tocopherol acetate is resistant to oxygen, since it lacks double bonds [3,9]. In the present study, the retention of niacin was more than 90% after 1 year of storage, which is consistent with Zhuge and Klopfenstein [19], who reported that the retention rate of VB_3_ during storage was 91% to 96% during 27 weeks storage. This was not surprising to us because VB_3_ has been reported to be the most stable among the B vitamins when added to feed or premixes [19] owing to its a stable molecular structure, which reduces its oxidation during storage. Coelho [8] reported that the loss of commercial fat-soluble vitamins after one month of storage was less than 10%; after six months of storage, its loss rate was 10% to 60%. Shurson et al [20] reported that vitamin activity in vitamin and VTM premixes decreased with prolonged storage time, but stability of VE, VB_2_, VB_3_, VB_5_, and VB_6_ are higher than that of other vitamins, which is in line with our results.

### Addition of choline chloride affected vitamin stability

Choline chloride has been reported to significantly affect vitamin activity [8–10]. Choline is considered a stress agent that affects vitamins that dissolve easily in water because it is hygroscopic, and can attract moisture to vitamin or VTM premixes [3]. The concentration of choline chloride in feed is usually higher than the micro-ingredient level, and problems related to both physical and chemical properties can be expected when choline is added to vitamin or VTM premixes [8–10]. After six months of storage, the loss of vitamin in a vitamin premix without choline chloride was 1% to 5% [8]; with choline chloride in the premix, the vitamin loss up to 32% after six months of storage [8]. In addition, the negative effects of choline chloride on vitamins in a VTM premix were more significant. After six months of storage, the loss of vitamin in a VTM premix without choline chloride was 12% to 30% [8]. However, in a VTM premix with choline chloride, the loss after six months was 23% to 52% [8]. Vitamin K is known for its contribution to the blood clotting or coagulation process. The VK_3_ was more stable in vitamin premix without choline than in the vitamin premix with choline during one-year storage, which was consistent with Tavčar-Kalcher and Vengušt [10]. Results from the present study suggest that loss of VK_3_ activity was approximately 60% in two vitamin premixes. Our results are similar to the average monthly losses reported by Coelho [8]. In the current study, choline chloride is a significant factor affecting the loss of VK_3_ activity. Menadione (VK_3_) is the form of vitamin K that is used in animal nutrition. It is not utilized in pure form in premix plants but was formulated with sodium bisulfite and derivatives. The most common menadione compound used in the industry is the water-soluble salt, menadione sodium bisulfite. It was reported that VK_3_ was very sensitive to moisture and trace minerals, and choline chloride was particularly destructive to VK_3_ [10]. Furthermore, supplemented choline chloride in vitamin or VTM premixes increased leaching of VK_3_ and prolonged oxidation-reduction reactions [9,10]. The inclusion of choline chloride in a VTM premix contributes to VA, VK_3_, VB_1_, and VB_2_ instability during storage. Stability data published by BASF 1994, cited by Whitehead [21], showed VA, VK_3_, VB_1_, and VB_2_ loss of 15%, 36%, 30%, and 5% after one month and 42%, 100%, 73%, and 44% after six months of storage in VTM premixes containing choline chloride. Besides, we observed that loss of VA, VK_3_, VB_1_, and VB_2_ was lower than that reported by Whitehead [21]. The reason may be that the vitamin manufacturing industry has developed products with improved stability. In the present study, we used thiamine mononitrate as VB_1_ source, it is used more often in feed because of its higher stability compared to thiamine hydrochloride [3,8]. In addition, the commercial form of VA and VB_2_ is spray-dried processing, which usually provides improved stability during storage and transportation.

### Excessive Cu and Zn elements in VTM premix affected vitamin stability

The VTM premixes are the most common dietary supplements. In commercial conditions, feeding piglets with high concentrations of Zn and Cu stimulates their average daily gain, decreases the feed conversion ratio, improves the digestibility of dietary nutrients and growth performance, and decreases the incidence of diarrhea [6,7]. However, minerals in premixes are usually in an inorganic form: sulfates, chlorides, oxides, etc. Different metal compounds have different capability of catalytic oxidation reaction [8]. In the present study, high levels of CuSO_4_ (more than 20,000 mg/kg of Cu in premix to promote growth) and ZnO (more than 225,000 mg/kg of Zn in premix to decrease the incidence of diarrhea) in VTM premixes resulted in the degradation of vitamins. Vitamin stability is reduced in the presence of certain trace minerals [8,9,20]. In our study, blending vitamins with trace minerals to form VTM premixes increased the loss of vitamin activity during prolonged storage periods. These trace minerals in premix can catalyze the generation of free radicals, thereby oxidizing antioxidants during storage. Certain metal-catalyzed destruction of vitamins in the feed matrix has been reviewed previously to a limited extent [22]. Trace minerals vary in their redox potential: Cu, Fe, and Zn are the most reactive, and Se, I, and Mg are less reactive minerals [8,22]. Reactive trace minerals reduce vitamin activity by oxidizing the vitamins. First, the metallic-like nature of trace minerals reduces the crystals of vitamins to smaller particles by eroding their protective coating. The smaller particles provide increased surface areas of vitamins for reactions between vitamin particles and trace mineral particles.

Dove and Ewan [23] reported that a high concentration of Cu (250 mg/kg feed) or Zn (1,000 mg/kg feed) increased vitamin loss. Redox-active transition metals, such as Cu, can serve as catalysts for the oxidation of organic compounds. Lu et al [24] reported that a high concentration of Cu sulfate promoted the undesirable oxidation of VE in feeds. Intriguingly, we did not find significant effects on VE content in the four VTM premixes during long-term storage. There are two significant factors that might contribute to achieve this positive characteristic. First, supplementation of VE are generally given in the form of all-rac-α-tocopheryl acetate in which the reactive hydroxyl group of α-tocopherol is esterified; rendering the molecule more stable than the free phenol form, and second, production of VE provides a physical barrier to reduce surface contact with pro-oxidant agents such as Zn or Cu. On the other hand, we observed long-term storage of VTM premix with high concentrations of Cu and Zn had no effect on VB_3_ and VB_5_ concentrations. The results from our study confirm previous studies on the stability of these vitamins. Shurson et al [20] reported recovery of VB_3_ after 120 days of storage in a vitamin stock was 96.68%, 86.04% in vitamin premix, and 87.04% in VTM premix. Zhuge and Klopfenstein [19] also reported that VB_3_ was considerably more stable than other vitamins; at the end of 27 weeks storage, VB_3_ in premixes with or without mineral retained 91% and 96%, respectively. The VB_3_ is probably the most stable of the water-soluble vitamins when added to feed or premixes, being little affected by heat, oxygen, moisture, or light [3,11]. However, there are no reports on the mechanism of resistance to degradation or subsequent degradation products. The VB_5_, pantothenic acid, is a constituent of coenzyme A, which both act as carriers of acyl groups and activators of carbonyl groups in many metabolic processes [2,3]. Pure VB_5_ is a viscous, hygroscopic, and chemically-unstable oil. In premixes, this vitamin is commonly added as calcium pantothenate, a soluble and stable solid. The calcium salt is preferred to the sodium salt, as solid forms of the sodium salt are much more hygroscopic. Similar to niacin, there were no significant influences of choline and high concentrations of Cu and Zn on pantothenic acid retention in VTM premixes. But storage time was a significant factor affecting the loss of VB_5_ under ambient temperature and relative humidity [3]. According to the previous results [20], there was no significant difference in the stability of VB_5_ after 120 days of storage at vitamin stock, vitamin premix and vitamin-inorganic trace minerals premix. A similar result was reported by Coelho [8]. Furthermore, it was reported that pantothenic acid was relatively stable to heat, oxygen, and light [8]. The stability of pantothenic acid is due to the presence of the carboxylic acid group to form two hydrogen bonds between a pair of molecules [25]. Furthermore, the amide group and two methyl groups in the aliphatic chain of pantothenic acid contribute to its stability.

In the current study, the supplementation of high concentrations of Cu and Zn significantly reduced the concentrations of VA, VD_3_, VK_3_, VB_1_, VB_2_, and VB_6_ in VTM premixes during storage. The reason can be explained that the presence of Zn and Cu in premix can speed up vitamin degradation [3,8, 9], these minerals can catalyze the generation of free radicals which can oxidize vitamins in VTM premixes. Also, the results from our study confirm previous studies on vitamin stability in VTM premix. Shurson et al [20] reported that the stability of VA, VK_3_, VB_1_, and VB_6_ in premixes was influenced by the presence of trace minerals. These results were in line with previous study. Yang et al [9] reported that stability of VA, VB_1_, and VB_6_ was affected by trace minerals, and the premixes containing high levels of Cu and Zn was more susceptible to oxidative reaction of vitamins. Factors that reduce VA activity are atmospheric oxygen, light, heat and other oxidizing agents [3]. Manan et al [26] looked at the stability of VA in the presence of Cu and Zn; the results showed that mineral fortification reduced VA stability by 36%. Pinkaew et al [27] reported the stability of VA in the presence of ZnO; the results showed that mineral fortification reduced the VA stability by 13.4%. The chemical structure of VA is an unsaturated monohydric alcohol with 20 carbon atoms, consisting of a cyclohexane ring linked to a polyunsaturated chain that terminates in an alcohol group. The five conjugated double bonds in the configuration of VA are easy points of attack for oxygen. The oxidation of the alcohol end group of VA results in the formation of retinal or all-trans retinaldehyde, which can be further oxidized to all-trans retinoic acid. Besides, the structure of VB_1_ can help to understand its instability during storage. The methylene bridge connecting the pyrimidine and thiazole moiety can easily be broken down by oxidizing ingredients [25]. In addition, the VB_6_ comprises a group of three related compounds: pyridoxine, pyridoxal, and pyridoxamine. Pyridoxine is commonly used for feed because pyridoxine is more stable than either pyridoxal or pyridoxamine [3,8], but pyridoxine was sensitive to light, particularly in neutral and alkaline solutions. The VB_6_ can lose bioactivity, particularly when minerals in the form of carbonates or oxides are present [8,11]. In the current study, the supplementation of high concentrations of Cu and Zn significantly reduced the concentrations of VB_6_ in VTM premixes during storage. The loss of VB_6_ activity after three months of storage at room temperature was 24% [11], which was slightly higher than VB_6_ loss in the present study. It may be that the degradation reaction of VB_6_ was enhanced by metal ions in the previous study. Loss of VB_6_ was lower when stored as vitamin premixes compared to VTM premixes, maybe the reason was that VTM premix contained more trace mineral. In VTM premixes, VB_6_ can lose bioactivity, particularly when minerals in the form of carbonates or oxides are present [8,11]. We used pyridoxine hydrochloride in the present trial, which is a commercially available form. And pyridoxine hydrochloride is the main supplement used in feed, because it has good handling properties and stability. In addition, Coelho [8] reported the loss of VB_6_ after six months of storage in a vitamin premix was 17%, and 32% in a VTM premix. Although the retention rate of VB_6_ in Coelho’s study [8] was completely inconsistent with our results, our data also show that the stability of VB_6_ is reduced in the premix containing inorganic trace minerals.

There are very limited stability data available for VD_3_ and VB_2_ in VTM premixes. The supplementation of high concentrations of Cu and Zn decreased the stability of VD_3_ and VB_2_ in VTM premixes in the present study. The VD_3_ is a fundamentally unstable compound containing double bounds that can be altered by different stresses and prone to degradation due to oxidation [3,28]. The stability of VD to oxidation and its instability to trace minerals were also reported by previous studies; Mahmoodani et al [28] demonstrated catalyzed isomerization of VD_3_ is liable *via* autoxidation to form a variety of oxidation products. Further, Zhuge and Klopfenstein [19] reported that VB_2_ was destroyed faster in the premix containing minerals and 54% of VB_2_ had been destroyed after 27 weeks of storage. In the last case, the loss of VB_2_ after six months of storage was 44% cited by Whitehead [21]. The most well-characterized aspect of riboflavin reactivity is its sensitivity to light in an aerobic environment which may be one of the reasons for low VB_2_ retention of previous studies. The rate of degradation can be promoted by complexation with some metal cations (e.g., Cu^2+^ and Zn^2+^) at the isoalloxazine moiety [29]. Vitamin A, VD_3_, VK_3_, VB_1_, VB_2_, and VB_6_ are relatively stable in vitamin premix, but degradation of vitamin was potentiated by the chemical reaction caused by the presence of Cu and Zn elements.

### Prediction equations for vitamin retention in premixes during storage

Prediction equations have been widely used to estimate values through regression analysis and can be a suitable proxy for conducting relevant experiments (i.e. loss in vitamin stability with time) and thus save time, reduce cost, and improve precision in diet formulation. To establish prediction equations of vitamin content in premix from storage time, regression analysis programming was used. The degradation of most of the vitamins followed first-order kinetics that can be developed [9,30]. The degradation rate accelerated progressively with storage time in vitamin and VTM premixes. What is more, VA, VK_3_, VB_1_, VB_2_, and VB_6_ degraded rapidly in VTM premix containing choline and high concentrations of Cu and Zn. When comparing the relative retention of vitamins, the data showed a significant deviation in different VTM premix. The VTM premix 4 has the most prominent sensitivity, followed by VTM premix 3 and 2. These observations are in line with previous report [9], showing poor vitamin retention in VTM premix containing choline and trace minerals. This differing behavior regarding vitamin degradation can be attributed to difference premix formulation and the presence of more metal ions, such as Cu^2+^ and Zn^2+^ that act as catalysts. The degradation of vitamins does not always increase linearly during storage, and the majority of predicted equations had good fit with the experimental data. This study is consistent with Giannakourou et al [30] who also found vitamin loss due to storage time was well predicted by exponential models. For prediction of vitamin loss during storage with non-linear kinetic models, this method has been proved effective to extract the kinetics parameters for vitamin degradation [31]. To the best of our knowledge, the degradation kinetics of vitamin in VTM premixes had not been studied before. The purpose of present study was to establish kinetic equations for vitamin retention in different premixes during storage. Validated kinetic models of vitamin for VTM premix, can be used for evaluation, control and proper management of the premix, with the application of suitable time indicators. Further, these results can serve for vitamin and VTM premixes production in order to improve the knowledge of vitamin its stability.

## CONCLUSION

The present study has provided information on the kinetics of vitamin degradation in different premixes during storage. The degradation of vitamins in all the samples under all storage conditions followed the first-order kinetics. Stability of VA, VK_3_, VB_1_, VB_2_, and VB_6_ was higher in VTM premixes containing no choline or high concentrations of Cu and Zn. The high concentration of trace minerals in the VTM premix can negatively affect VD_3_ stability. Our study also suggested that considering the potential losses of vitamins in formulation, the time between the manufacturing and use of the vitamin premixes should be minimized, and vitamins and trace mineral premixes should be stored separately. This work could be used as a guideline for the fortification of vitamin and VTM premixes.

## Figures and Tables

**Table 1:** Composition of the vitamin and vitamin-trace mineral premixes1)

Item	Vitamin premix 1	Vitamin premix 2	VTM premix 1	VTM premix 2	VTM premix 3	VTM premix 4
Vitamin2) (unit/kg)						
VA (IU)	5,400,000	5,400,000	1,350,000	1,350,000	1,350,000	1,350,000
VD_3_ (IU)	1,200,000	1,200,000	300,000	300,000	300,000	300,000
VE (IU)	12,000	12,000	3,000	3,000	3,000	3,000
VK_3_ (mg)	1,200	1,200	300	300	300	300
VB_1_ (mg)	1,200	1,200	300	300	300	300
VB_2_ (mg)	2,400	2,400	600	600	600	600
VB_3_ (mg)	12,000	12,000	3,000	3,000	3,000	3,000
VB_5_ (mg)	7,200	7,200	1,800	1,800	1,800	1,800
VB_6_ (mg)	1,200	1,200	300	300	300	300
Folic acid (mg)	48	48	12	12	12	12
Biotin (mg)	12	12	3	3	3	3
Vitamin B_12_ (mg)	9.6	9.6	2.4	2.4	2.4	2.4
Choline (mg)	-	160,000	-	40,000	-	40,000
Trace mineral3) (mg/kg)						
Cu	-	-	500	500	20,000	20,000
I	-	-	14	14	14	14
Fe	-	-	10,000	10,000	10,000	10,000
Mg	-	-	300	300	300	300
Se	-	-	25	25	25	25
Zn	-	-	8,000	8,000	225,000	225,000

VTM, vitamin-trace mineral; VA, vitamin A; VD_3_, vitamin D_3_; VE, vitamin E; VK_3_, vitamin K_3_; VB_1_, vitamin B_1_; VB_2_, vitamin B_2_; VB_3_; vitamin B_3_; VB_5_, vitamin B_5_; VB_6_, vitamin B_6_.

1) Vitamin premixes were designed to be added at a rate of 2.5 g/kg of the diet. The VTM premixes were designed to be added at a rate of 10 g/kg of the diet. Vitamin premix 1 contained no choline chloride, Vitamin premix 2 contained 160,000 mg/kg of choline chloride. VTM premix 1 contained 500 mg/kg of CuSO_4_ and 8,000 mg/kg of ZnO, VTM premix 2 contained 40,000 mg/kg of choline chloride, 500 mg/kg of CuSO4 and 8,000 mg/kg of ZnO. VTM premix 3 contained 20,000 mg/kg of CuSO_4_ and 225,000 mg/kg of ZnO. VTM premix 4 contained 40,000 mg/kg of choline chloride, 20,000 mg/kg of CuSO_4_ and 225,000 mg/kg of ZnO. Limestone and rice by-product as carriers mix purified forms of vitamins and trace minerals to achieve the pre-determined diet inclusion rate.

2) Vitamin sources: VA, retinyl acetate; VD_3_, cholecalciferol; VE, D, L-α-tocopherol acetate; VK_3_, menadione sodium bisulfite; VB_1_, thiamine mononitrate; VB_2_, riboflavin; VB_3_, nicotinic acid; VB_5_, D-calcium pantothenate; VB_6_, pyridoxine hydrochloride; VB_7_, biotin; Vitamin B_9_, folic acid; Vitamin B_12_, cyanocobalamin; choline, choline chloride.

3) Trace mineral source: Cu, CuSO_4_; I, Ca(IO_3_)_2_; Fe, FeSO_4_; Mg, MnO; Se, NaSeO_2_; Zn, ZnO.

**Table 2:** Analyzed initial values of vitamin in vitamin and vitamin-trace mineral premixes

Item	Vitamin premix 1	Vitamin premix 2	VTM premix 1	VTM premix 2	VTM premix 3	VTM premix 4
Vitamin1) (unit/kg)						
VA (IU)	5,317,420	5,350,883	1,367,333	1,393,358	1,358,165	1,382,106
VD_3_ (IU)	1,169,405	1,197,127	300,297	302,709	301,525	303,454
VE (IU)	12,044	11,996	2,942	2,942	2,963	2,979
VK_3_ (mg)	1,171	1,164	297	306	294	293
VB_1_ (mg)	1,212	1,179	303	302	299	307
VB_2_ (mg)	2,436	2,487	585	597	589	601
VB_3_ (mg)	12,167	12,015	3,017	3,019	2,984	3,018
VB_5_ (mg)	7,173	7,226	1,836	1,785	1,768	1,847
VB_6_ (mg)	1,202	1,191	302	296	298	306
Choline (mg)	-	166,713	-	44,333	-	43,448

Values represent means of six replicate samples each analyzed in duplicate.

VTM premix, vitamin-trace mineral premix; VA, vitamin A; VD_3_, vitamin D_3_; VE, vitamin E; VK_3_, vitamin K_3_; VB_1_, vitamin B_1_; VB_2_, vitamin B_2_; VB_3_; vitamin B_3_; VB_5_, vitamin B_5_; VB_6_, vitamin B_6_.

1) Vitamin premix 1 contained no choline chloride, Vitamin premix 2 contained 160,000 mg/kg of choline chloride. VTM premix 1 contained 500 mg/kg of CuSO_4_ and 8,000 mg/kg of ZnO, VTM premix 2 contained 40,000 mg/kg of choline chloride, 500 mg/kg of CuSO4 and 8,000 mg/kg of ZnO. VTM premix 3 contained 20,000 mg/kg of CuSO_4_ and 225,000 mg/kg of ZnO. VTM premix 4 contained 40,000 mg/kg of choline chloride, 20,000 mg/kg of CuSO_4_ and 225,000 mg/kg of ZnO.

**Table 3:** Probabilities of interactive and main effects of storage time and choline on stability (as defined by percentage of initial vitamin activity) of vitamins in vitamin premixes

Item	Interactive effects	Main effects	
	
	Time×choline	Time	Choline
VA	0.628	0.096	0.076
VD_3_	0.644	<0.001	0.958
VE	0.987	0.957	0.433
VK_3_	<0.001	<0.001	<0.001
VB_1_	0.962	<0.001	0.273
VB_2_	0.949	<0.001	0.742
VB_3_	0.934	0.493	0.111
VB_5_	0.999	<0.001	0.053
VB_6_	0.557	<0.001	0.898

VA, vitamin A; VD_3_, vitamin D_3_; VE, vitamin E; VK_3_, vitamin K_3_; VB_1_, vitamin B_1_; VB_2_, vitamin B_2_; VB_3_; vitamin B_3_; VB_5_, vitamin B_5_; VB_6_, vitamin B_6_.

**Table 4:** Probabilities of interactive and main effects of storage time, choline, and Cu and Zn elements on stability (as defined by percentage of initial vitamin activity) of vitamins in vitamin-trace mineral premixes

Item	VA	VD_3_	VE	VK_3_	VB_1_	VB_2_	VB_3_	VB_5_	VB_6_
Interactive effects									
Time×choline×Cu/Zn	0.310	0.868	0.680	0.002	0.013	0.009	0.984	0.993	0.654
Time×choline	0.011	0.969	0.899	<0.001	<0.001	<0.001	0.568	0.973	0.052
Time×Cu/Zn	<0.001	<0.001	0.320	<0.001	<0.001	0.001	0.729	0.993	<0.001
Choline×Cu/Zn	0.075	0.316	0.925	<0.001	0.603	0.653	0.846	0.500	0.104
Main effects									
Time	<0.001	<0.001	<0.001	<0.001	<0.001	<0.001	<0.001	<0.001	<0.001
Choline	<0.001	0.404	0.812	<0.001	<0.001	<0.001	0.886	0.483	0.848
Cu/Zn	<0.001	<0.001	0.970	<0.001	<0.001	<0.001	0.937	0.740	<0.001

VA, vitamin A; VD_3_, vitamin D_3_; VE, vitamin E; VK_3_, vitamin K_3_; VB_1_, vitamin B_1_; VB_2_, vitamin B_2_; VB_3_; vitamin B_3_; VB_5_, vitamin B_5_; VB_6_, vitamin B_6_.

**Table 5:** Prediction equations for vitamin retention (%) in different vitamin-trace mineral premixes during storage

No.	Item	Types1)	Predicted equations2)	R^2^	RMSEP	p-value
1	VA	VTM premix 1	y = −5.706x+106.014	0.998	1.158	<0.001
2	VA	VTM premix 2	y = −8.967x+106.085	0.974	7.573	0.002
3	VA	VTM premix 3	y = 144.432e^−0.286x^	0.989	0.151	<0.001
4	VA	VTM premix 4	y = 159.533e^−0.503x^	0.882	0.944	0.018
5	VD_3_	VTM premix 1	y = 100.97e^−0.025x^	0.977	0.020	0.002
6	VD_3_	VTM premix 2	y = 102.152e^−0.027x^	0.985	0.017	0.001
7	VD_3_	VTM premix 3	y = 98.338e^−0.041x^	0.919	0.063	0.010
8	VD_3_	VTM premix 4	y = 95.57e^−0.039x^	0.900	0.066	0.014
9	VE	VTM premix 1	y = 98.111e^−0.008x^	0.797	0.020	0.042
10	VE	VTM premix 2	y = 98.505e^−0.008x^	0.944	0.010	0.006
11	VE	VTM premix 3	y = 97.331e^−0.006x^	0.756	0.017	0.055
12	VE	VTM premix 4	y = 97.288e^−0.006x^	0.554	0.026	0.146
13	VK_3_	VTM premix 1	y = 103.721e^−0.058x^	0.992	0.028	<0.001
14	VK_3_	VTM premix 2	y = 101.025e^−0.068x^	0.945	0.085	0.006
15	VK_3_	VTM premix 3	y = 108.391e^−0.087x^	0.989	0.047	0.001
16	VK_3_	VTM premix 4	y = 112.062e^−0.152x^	0.975	0.125	0.002
17	VB_1_	VTM premix 1	y = 102.21e^−0.054x^	0.970	0.042	0.001
18	VB_1_	VTM premix 2	y = 98.939e^−0.094x^	0.799	0.240	0.041
19	VB_1_	VTM premix 3	y = 99.533e^−0.081x^	0.876	0.157	0.019
20	VB_1_	VTM premix 4	y =133.472e^−0.242x^	0.992	0.095	<0.001
21	VB_2_	VTM premix 1	y = 97.178e^−0.01x^	0.810	0.026	0.037
22	VB_2_	VTM premix 2	y = 90.3e^−0.012x^	0.492	0.062	0.187
23	VB_2_	VTM premix 3	y = 97.393e^−0.003x^	0.477	0.018	0.217
24	VB_2_	VTM premix 4	y = 94.924e^−0.015x^	0.861	0.031	0.023
25	VB_3_	VTM premix 1	y = 98.349e^−0.004x^	0.838	0.009	0.029
26	VB_3_	VTM premix 2	y = 99.114e^−0.005x^	0.904	0.009	0.013
27	VB_3_	VTM premix 3	y = 99.292e^−0.006x^	0.774	0.016	0.049
28	VB_3_	VTM premix 4	y = 99.252e^−0.006x^	0.896	0.010	0.015
29	VB_5_	VTM premix 1	y = 100.996e^−0.006x^	0.884	0.012	0.017
30	VB_5_	VTM premix 2	y = −0.672x+101.276	0.887	1.231	0.017
31	VB_5_	VTM premix 3	y = −0.647x + 100.34	0.907	0.908	0.008
32	VB_5_	VTM premix 4	y = −0.62x + 101.288	0.913	0.840	0.007
33	VB_6_	VTM premix 1	y = −1.946x + 101.076	0.998	0.437	<0.001
34	VB_6_	VTM premix 2	y = 101.315e^−0.018x^	0.984	0.012	0.001
35	VB_6_	VTM premix 3	y = 101.667e^−0.027x^	0.810	0.067	0.037
36	VB_6_	VTM premix 4	y = 97.419e^−0.02x^	0.747	0.060	0.059

VTM, vitamin-trace mineral; RMSEP, root mean square error of prediction; VA, vitamin A; VD_3_, vitamin D_3_; VE, vitamin E; VK_3_, vitamin K_3_; VB_1_, vitamin B_1_; VB_2_, vitamin B_2_; VB_3_; vitamin B_3_; VB_5_, vitamin B_5_; VB_6_, vitamin B_6_.

1) VTM premix 1 contained 500 mg/kg of CuSO_4_ and 8,000 mg/kg of ZnO; VTM premix 2 contained 40,000 mg/kg of choline chloride, 500 mg/kg of CuSO_4_ and 8,000 mg/kg of ZnO; VTM premix 3 contained 20,000 mg/kg of CuSO_4_ and 225,000 mg/kg of ZnO; VTM premix 4 contained 40,000 mg/kg of choline chloride, 20,000 mg/kg of CuSO_4_ and 225,000 mg/kg of ZnO.

2) y (%) is retention of vitamin, x (month) is storage time.
